# Porcine fungal mock community analyses: Implications for mycobiome investigations

**DOI:** 10.3389/fcimb.2023.928353

**Published:** 2023-02-08

**Authors:** Ann M. Arfken, Juli Foster Frey, Nora Isabel Carrillo, Nneka Ijeoma Dike, Ogechukwu Onyeachonamm, Daniela Nieves Rivera, Cary Pirone Davies, Katie Lynn Summers

**Affiliations:** ^1^ Oak Ridge Institute for Science and Education, Center for Disease Control, Atlanta, GA, United States; ^2^ Animal Biosciences and Biotechnology Laboratory, Beltsville Agricultural Research Center, Agricultural Research Service, United States Department of Agriculture, Beltsville, MD, United States

**Keywords:** fungi, mock community, microbiome, mycobiome, swine, pig, *Kazachstania slooffiae*

## Abstract

**Introduction:**

The gut microbiome is an integral partner in host health and plays a role in immune development, altered nutrition, and pathogen prevention. The mycobiome (fungal microbiome) is considered part of the rare biosphere but is still a critical component in health. Next generation sequencing has improved our understanding of fungi in the gut, but methodological challenges remain. Biases are introduced during DNA isolation, primer design and choice, polymerase selection, sequencing platform selection, and data analyses, as fungal reference databases are often incomplete or contain erroneous sequences.

**Methods:**

Here, we compared the accuracy of taxonomic identifications and abundances from mycobiome analyses which vary among three commonly selected target gene regions (18S, ITS1, or ITS2) and the reference database (UNITE - ITS1, ITS2 and SILVA - 18S). We analyze multiple communities including individual fungal isolates, a mixed mock community created from five common fungal isolates found in weanling piglet feces, a purchased commercial fungal mock community, and piglet fecal samples. In addition, we calculated gene copy numbers for the 18S, ITS1, and ITS2 regions of each of the five isolates from the piglet fecal mock community to determine whether copy number affects abundance estimates. Finally, we determined the abundance of taxa from several iterations of our in-house fecal community to assess the effects of community composition on taxon abundance.

**Results:**

Overall, no marker-database combination consistently outperformed the others. Internal transcribed space markers were slightly superior to 18S in the identification of species in tested communities, but *Lichtheimia corymbifera*, a common member of piglet gut communities, was not amplified by ITS1 and ITS2 primers. Thus, ITS based abundance estimates of taxa in piglet mock communities were skewed while 18S marker profiles were more accurate. *Kazachstania slooffiae* displayed the most stable copy numbers (83-85) while *L. corymbifera* displayed significant variability (90-144) across gene regions.

**Discussion:**

This study underscores the importance of preliminary studies to assess primer combinations and database choice for the mycobiome sample of interest and raises questions regarding the validity of fungal abundance estimates.

## Introduction

The mycobiome is an often overlooked, but critical component in animal health (Cui et al., 2013; Huffnagle and Noverr, 2013; Huseyin et al., 2017; Enaud et al., 2018; Chin et al., 2020; Summers and Arfken 2022). While studies investigating the bacteriome (bacterial microbiome) have become prevalent in the literature, studies involving fungal populations are less common. Amplicon-based workflows, which employ next generation sequencing, are commonly used for fungal profiling, but there is no current consensus over which methods are optimal. Challenges associated with diverse fungal cell wall structures, similarity of conserved fungal marker regions with those of other eukaryotes, and sparse or inaccurate reference databases lead to biases during sample collection/storage, DNA extraction, marker and primer selection, sequencing, and bioinformatic analyses (Henrik et al., 2008; Nilsson et al., 2019). Thus, technical studies investigating potential confounding factors are vital in pushing this field forward.

The selection of an appropriate gene marker is a critical aspect of amplicon-based mycobiome profiling. Markers may differ in length, taxonomic resolving power, and ease with which they amplify different species, all of which affect taxonomic classifications (Begerow et al., 2010; Berruti et al., 2017; Banos et al., 2018; George et al., 2019). The Internal Transcribed Spacer (ITS) of the rRNA ribosomal gene is most frequently used for fungal barcoding. The ITS sequences are highly variable and provide species level resolution of diverse taxa (Schubert et al., 2007; Nilsson et al., 2008; Schoch et al., 2012; Lücking et al., 2020). However, ITS may not differentiate some species, such as those from the genus *Fusarium* (Karlsson et al., 2017; Walder et al., 2017; Bakker, 2018), or some molds and pathogens (Lücking et al., 2020), and this region is only useful when closely related sequences are present in the database (Eldred et al., 2021). Further, variability in ITS length can confound taxonomic identifications and abundance estimates (Lindahl et al., 2013; Tedersoo et al., 2015; Tedersoo and Lindahl, 2016; Reich and Labes, 2017). Limitations in Illumina sequencing preclude sequencing the entire ITS region, and there is debate as to whether the ITS1 or ITS2 region is best (Nilsson et al., 2008).

The 18S region of rRNA is also commonly employed for fungal community profiling (Raja et al., 2017b; Reich and Labes, 2017; Frau et al., 2019). This region is highly conserved and valued for its ability to resolve phylogenetic relationships at high taxonomic levels (Begerow et al., 2010; Schoch et al., 2012). Although ITS is generally preferred for species level profiling (Schoch et al., 2012; Nilsson et al., 2019), in some taxonomic groups,18S can better resolve species than ITS (Berruti et al., 2017; Frau et al., 2019), and in some cases, community diversity measures may be comparable using 18S or ITS (George et al., 2019).

The selection of a reference database is also a major factor in the identification of species. The development of fungal databases has lagged behind those of bacteria, and current databases are often incomplete or erroneous (Nilsson et al., 2019). Sequence errors, or the lack of sequences for some taxonomic groups, can skew taxonomic identifications which are based on the similarity between amplicon and reference sequences. Errors can occur in both database and experimental sequences due to polymerase mistakes, chimera formation, and incorrect base-calling (Amend et al., 2010; Lindahl et al., 2013; Gohl et al., 2016; Tedersoo and Lindahl, 2016; Bakker, 2018). In addition, sequences that are labeled with the incorrect taxonomic assignments can further confound classifications (Schoch et al., 2012; Glockner et al., 2017; Yarza et al., 2017; Nilsson et al., 2019) and complications surrounding the identification of sexual and asexual forms of fungi contribute to these errors (Halwachs et al., 2017). Further, classification algorithms which compare experimental and database sequences may also skew results (Wang et al., 2007). Currently, the largest collection of 18S and ITS sequences are found in the SILVA (Quast et al., 2013) and UNITE (Nilsson et al., 2019) databases, respectively.

In order enhance the accuracy of mycobiome studies in agricultural animals, we assessed the cumulative impact of experimental biases on identification and quantification accuracy through Illumina MiSeq sequencing with three different workflows performed on synthetic and in-house constructed mock communities. We utilized combinations of three gene regions (18S, ITS1, ITS2) and analyzed each with either the SILVA or UNITE databases: 18S-SILVA, ITS1-UNITE, and ITS2-UNITE. We then determined how taxonomic identifications and abundance estimates varied among workflows. Communities included in-house constructed piglet fecal mock fungal communities (Isolate and Mixed), a piglet fecal community (Fecal), and a commercially available fungal mock community (ATCC Reference Standard). We also estimated the number of gene copies of 18S, ITS1, and ITS2 present in each member of the piglet fecal mock community as copy numbers of chromosomes vary and the number of ITS and 18S regions vary significantly among species, and even strains, in fungi (Herrera et al., 2009; Black et al., 2013; Steenwyk and Rokas, 2018; Lofgren et al., 2019), and high numbers of the target marker gene can lead to an over-estimation of certain species or strains. These findings help set a framework for experimental design and considerations when investigating the mycobiome in porcine and other agricultural samples.

## Materials and methods

This study utilized a combination of environmental and commercial samples to investigate four defined mock fungal communities: (1) Isolate, (2) Mixed, (3) Fecal, and (4) ATCC Reference Standard. Below are the details on the construction of each community type.

### Piglet fecal sample collection

This animal study was reviewed and approved by the USDA-ARS Institutional Animal Care and Use Committee of the Beltsville Agricultural Research Center. No antibiotics, antifungals, or supplementary additives were administered to the piglets at any time during the experiment. The diet was formulated to meet the National Research Council estimate of nutrient requirements (**Supplemental Table 1**). Piglets were weaned at 21 days of age and fecal samples were collected from 6 post-weaning piglets (age 24d) using sterile cotton-tipped swabs (Puritan, Guilford, ME, USA) to stimulate defecation into sterile weigh basins. Fecal samples were transferred to sterile 50 mL conical tubes and transported back to the laboratory directly.

### Construction of isolate and mixed mock communities derived from piglet feces

**Figure 1:**
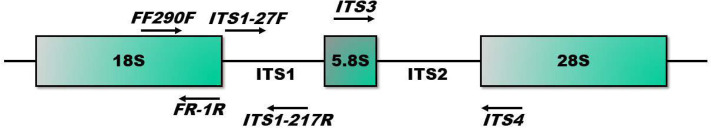
ITS1, ITS2, and 18S primer targets tested. Primers targeting ITS1(ITS1-27F, ITS1-217R), ITS2 (ITS3, ITS4), and 18S (FF290F, FR-1R) were assessed for amplicon accuracy.

Two mL of sterile 1X PBS was added to 0.2 g of collected feces and homogenized in a biological safety cabinet with a tissue tearer (Omni International, Kennesaw, GA, USA) and sterilization steps between each sample. Post-homogenization, samples were serially diluted and plated in triplicate on Sabouraud Dextrose Agar (SDA) and Yeast Potato Dextrose Agar (YPD) plates (BD Difco, Franklin Lakes, NJ, USA). Both agar types were supplemented with 0.1 mg/ml cefoperazone, a third-generation broad-spectrum cephalosporin, to reduce bacterial growth on the agar plates (Sigma-Aldrich, St. Louis, MO, USA). Agar plates were incubated under different temperature conditions (37° C, 20° C) and with and without 5% CO_2_ supplementation to optimize growth for multiple fungi. Each fungal isolate was preliminarily identified under a phase contrast microscope to assess the presence or absence of year cells (shape, size), conidia presence (size, shape), or hyphae (septate or aseptate, branching or not, pigment presence, and width evenness). These assessments were utilized in combination with sequencing results to confirm identification. Colonies that grew and were assessed under the microscope were then individually Sanger sequenced utilizing primers for the ITS1, ITS2, and 18S regions in fungi (**Table 1** and **Figure 1**). ITS1 primers refer to: ITS1 Forward – 5’ CTTGGTCATTTAGAGGAAGTCC 3’, ITS1 Reverse – 5’ GCTGCGTTCTTCATCGATGC 3’. ITS2 primers refer to: ITS2 Forward – 5’ GCATCGATGAAGAACGCAGC 3’, ITS2 Reverse – 5’ TCCTCCGCTTATTGATATGC 3’. 18S primers refer to: 18S Forward – 5’ CGATAACGAACGAGACCT 3’, 18S Reverse – 5’ ANCCATTCAATCGGTANT 3’. Sequences were identified by comparison to the nr/nt database using BLAST (blast.ncbi.nlm.nih.gov) (pident >99%, qcov=100).

**Table 1:** Primers utilized in amplifying fungi.

Primer Target	Primer Name	Primer Sequence (5’ – 3’)	Citation
**ITS1 Forward**	ITS1F	CTTGGTCATTTAGAGGAAGTAA	(Usyk et al., 2017)
**ITS1 Reverse**	ITS2	GCTGCGTTCTTCATCGATGC	(Usyk et al., 2017)
**ITS2 Forward**	ITS3	GCATCGATGAAGAACGCAGC	(White et al., 1990)
**ITS2 Reverse**	ITS4	TCCTCCGCTTATTGATATGC	(White et al., 1990)
**18S Forward**	FF390F	CGATAACGAACGAGACCT	(Vainio and Hantulo, 2000)
**18S Reverse**	FR1R	ANCCATTCAATCGGTANT	(Vainio and Hantulo, 2000)

Five fungal isolates identified from piglet feces were utilized for mock community construction and further analysis: *Kazachstania slooffiae* (K), *Trichosporon asahii* (T), *Pichia fermentans* (P), *Lichtheimia corymbifera* (L), and *Candida albicans* (C). In brief, colonies of each identified species were cultured in 5 mL of YPD+cef or SDA+cef. After 24 h of growth at 37° C and 5% CO_2_, whole DNA was isolated using the DNeasy PowerSoil Pro Kit according to Qiagen protocol (Qiagen, Hilden, Germany) with the addition of mechanical bead beating for 20 min at 20 frequency/second. Negative extraction controls were run by incorporating 1X sterile PBS at the beginning of isolation instead of a biological sample. Single isolate communities were made from each of the 5 single isolates with 125 ng DNA in each PCR reaction (Isolate Community). From each isolate, combinations of the fungal isolates were used to create mixed mock communities ranging in complexity from two isolates up to five isolates (Mixed Mock Community). These mixed mock communities included equivalent DNA from each included isolate (**Table 2**).

**Table 2:** ITS1, ITS2, and 18S sequencing results for fungal mock communities.

Sample Description	ITS1	ITS2	18S
	Sequences	Sequences	Sequences
ATCC Reference Standard Plate 1A	216,347	151,428	29,926
ATCC Reference Standard Plate 1B	281,004	143,514	151*
ATCC Reference Standard Plate 1C	268,660	100,076	104,724
ATCC Reference Standard Plate 2A	873,647	199,553	27,778
ATCC Reference Standard Plate 2B	525,129	172,878	96,266
ATCC Reference Standard Plate 2C	115,200	116,659	73,825
C triplicate 1	321,844	110,226	66,710
C triplicate 2	302,379	105,032	136,926
C triplicate 3	334,917	138,004	154,817
K triplicate 1	197,052	102,368	80,883
K triplicate 2	175,115	151,050	109,118
K triplicate 3	240,375	59,111	91,949
L triplicate 1	103,287	46,674	84,989
L triplicate 2	123,107	62,931	114,244
L triplicate 3	75,192	74,567	108,347
P triplicate 1	282,184	201,596	143,021
P triplicate 2	449,849	85,021	41,668
P triplicate 3	291,850	129,177	134,574
T triplicate 1	201,244	157,752	81,949
T triplicate 2	228,066	182,574	100,822
T triplicate 3	258,368	138,499	59,965
KCTPL triplicate 1	138,282	130,129	110,821
KCTPL triplicate 2	292,101	169,426	125,302
KCTPL triplicate 3	210,269	110,507	102,825
KC triplicate 1	136,187	132,536	96,232
KC triplicate 2	139,152	141,500	97,744
KC triplicate 3	152,675	94,197	144,150
KT triplicate 1	246,612	115,898	116,841
KT triplicate 2	233,873	130,638	111,852
KT triplicate 3	232,672	156,547	112,806
D24 triplicate 1	8,413	142,635	23,292
D24 triplicate 2	7,305	104,004	29,260
D24 triplicate 3	7,307	74,674	47,743
KP triplicate 1	139,972	162,227	98,435
KP triplicate 2	177,714	138,624	77,041
KP triplicate 3	168,201	165,831	109,204
KL triplicate 1	75,977	64,326	158,573
KL triplicate 2	49,379	88,947	195,678
KL triplicate 3	67,565	72,994	237,263
KCT triplicate 1	206,400	209,313	87,890
KCT triplicate 2	146,795	151,530	122,291
KCT triplicate 3	166,199	238,977	143,523
KCL triplicate 1	267,948	324,762	179,015
KCL triplicate 2	220,305	352,179	119,578
KCL triplicate 3	229,932	195,562	138,472
KTP triplicate 1	378,878	62,507	53,611
KTP triplicate 2	247,084	239,078	96,828
KTP triplicate 3	362,214	223,709	84,090
KTL triplicate 1	282,468	191,181	90,063
KTL triplicate 2	375,484	199,343	71,909
KTL triplicate 3	292,127	77,309	119,594
KPL triplicate 1	297,558	193,837	125,864
KPL triplicate 2	174,932	79,975	95,827
KPL triplicate 3	222,200	140,314	112,623
KCP triplicate 1	238,044	145,369	75,786
KCP triplicate 2	161,580	134,908	78,079
KCP triplicate 3	403,014	172,619	75,374
*Excluded from analysis			
AVERAGE	228,450	143,102	101,928

Sample descriptions with sequence results are shown for each community and primer combination. Here the acronyms represent the fungal isolate: C, Candida; K, Kazachstania; L, Lichtheimia; P, Pichia; and T, Trichosporon. The sequencing results from each of the isolate triplicates is listed for each primer pair.

C, Candida albicans; K, Kazachstania slooffiae; L, Lichtheimia corymbifera; P, Pichia fermentans; T, Trichosporon asahii; D24, Day 24 Feces.

### Fecal and ATCC Reference Standard mock communities

6 piglet (d24) feces were sterilely collected from piglets (as above) and 250mg was used to prepare DNA for MiSeq to represent a typical fecal sample used in a microbiome study (Fecal Community). The DNA was isolated as above utilizing the DNeasy PowerSoil Pro Kit (Qiagen, Hilden, Germany) with the same parameters as the isolates. A commercial mycobiome genomic DNA mix from ATCC (MSA 1010) (ATCC, Manassas, VA, USA) was also included in this study to serve as a positive control (ATCC Reference Standard). This community is composed of genomic DNA from diverse environmental fungi at known concentrations (**Table 3**). Technical replicates of the commercial community were named “ATCC Reference Standard 1” and “ATCC Reference Standard 2”.

**Table 3:** Composition of fungal genomic DNA found in ATCC MSA 1010 mix (ATCC Reference Standard).

Composition	Fungal isolate
10%	*Aspergillus fumigatus* (ATCC MYA-4609D-5)
10%	*Cryptococcus neoformans* var. *grubli* (ATCC 208821D-2)
10%	*Trichophyton interdigitale* (ATCC 9533D-5)
10%	*Penicillium chrysogenum* (ATCC 10106D-5)
10%	*Fusarium keratoplasticum* (ATCC 36031D-5)
10%	*Candida albicans* (ATCC 10231D-5)
10%	*Candida glabrata* (ATCC 2001D-5)
10%	*Malassezia globose* (ATCC MYA-4612D-5)
10%	*Saccharomyces cerevisiae* (ATCC 201390D-5)
10%	*Cutaneotrichosporon dermatis* (ATCC 204094D-5)

### ITS1, ITS2, and 18S amplification, and Illumina Sequencing

For each of the four different community types: (1) Isolate, (2) Mixed, (3) Fecal, and (4) ATCC Reference Standard, the ITS1, ITS2, and 18S regions were amplified in triplicate using gene specific primers listed in **Table 1** with the Illumina adaptor sequence added to the 5’ end (5′-TCGTCGGCAGCGTCAGATGTG TATAAGAGACAG—ITS3-3′) and (5′GCTTCGTGGGCTCGGAGATGTGTATAAG AGACAG—ITS4-3′). The Illumina 16S Metagenomic Sequencing Library Preparation protocol was utilized and samples were sequenced on the Illumina MiSeq platform (https://support.illumina.com/documents/documentation/chemistry_documentation/16s/16s-metagenomic-library-prep-guide-15044223-b.pdf).

### Experimental controls

To assess contamination during DNA extraction and sequencing, and to detect biases in database identifications, we implemented several controls. Negative controls were included at each step of extraction (see above) and sequencing (wells with no template) to track any contamination. The ATCC mock community served as a positive control. As all samples were unable to be sequenced on one Illumina MiSeq sequencing run, cross-plate samples for ITS and 18S targets served as internal controls to assess plate sequencing efficiency. No significant difference in sequencing efficiency was found between plates (data not shown). Samples were run in technical triplicate for each community type and those utilized as cross-plate controls were run in triplicate on each plate and duplicate on comparison plates.

### Illumina sequence processing and taxonomic identification

Forward and reverse primers were removed from paired end reads using cutadapt v 1.18 for ITS sequences and BBDuk in BBTools v 38.79 for 18S sequences (Martin, 2011). Trimmomatic v 0.39 and the SLIDINGWINDOW 4:15 and MINLEN:40 options were used to quality trim individual sequences for all three data sets (Bolger et al., 2014). Merging, denoising, chimera removal and amplicon sequence variant (ASV) determination and ASV sequence lengths for each data set was conducted using the DADA2 plugin in QIIME2 v 2020.8 with truncation and trimming parameters set to 0. Taxonomic databases for classification were trained using a Naïve Bayes classifier for ITS and 18S with the q2-feature-classifier commands in QIIME2 using the developer’s QIIME-formatted UNITE v8 2020.4 full-length dynamic dataset and the QIIME-formatted SILVA 138 SSURef NR99 full-length dataset (Bokulich et al., 2020), respectively. ASVs that were classified as either “unclassified” or “unidentified” at the phylum level were searched against the NCBI nt database using BLAST and reclassified as either “Fungi Unclassified” or “Non-Fungi” or discarded (no hit or query coverage and percent identity < 80%). Except for negative controls, samples < 5000 sequences were removed from analysis (ITS1 n=0; ITS2 n=0; 18S n = 1). All sequences are publicly available (BioProject PRJNA693350).

### Copy number determination by qPCR

The 18S, ITS1, and ITS2 regions of each of the five fungal mock community species were first amplified and sequenced. Each qPCR primer set was designed for the ITS1, ITS2, and 18S regions of all 5 fungal mock community species from these sequences (**Supplemental Table 2**). The qPCR primers were also designed for the actin gene of all 5 mock community species, using GenBank sequences as reference. Preliminary studies were done to assess standard curves and melting points for each of the 4 primer sets for each fungal species (**Supplemental Table 4**). The Primer 3 program (https://bioinfo.ut.ee/primer3-0.4.0/) was used to assist with qPCR primer design, and trial PCR reactions were performed using the newly designed PCR primers to ensure a single product of the predicted size. All PCR products were between 108-150 bp. qPCR was performed in triplicate on 6 different concentrations of DNA template ranging from 0-25 ng for each of the five fungal species, using actin, ITS1, ITS2, and 18S primers. SsoAdvanced Universal SYBR Green Supermix (BioRad) was used and primers had a final concentration of 0.5 µM. The qPCR program was as follows: 95˚ C 3 min, followed by 40 cycles of 95˚ C 10 sec and 60˚ C 30 sec. A melt curve of 60-95˚ C at 0.5˚ C was performed. Calculations were performed as previously published (Bakker, 2018) to determine the range of gene copy number. Ribosomal RNA gene copy number within the genome was estimated as: Copy number = 2 ^[C(t)^_single copy_^-C(t)^_rRNA_^].^

## Results

### Amplicon sequencing outcomes for ITS1, ITS2, and 18S gene targets

Gene targets, ITS1, ITS2, and 18S were separately amplified and sequenced in triplicate from 19 communities including 5 Isolates, 11 Mixed, 1 Fecal, and 2 ATCC Reference Standards for a total of 171 samples (**Table 2**). Sequence outputs ranged from 7,305 to 873,647 total read pairs (**Table 2**). One replicate (of triplicates) for the ATCC Reference Standard community was excluded from the 18S sequencing library for further analysis due to low sequencing yield (n = 151 reads; **Table 2**). The ITS1 libraries resulted in the highest number of total paired reads (1.30x10^7^) with an average read depth per sample of 228,450 ± 18,226. The ITS2 libraries resulted in 8.16x10^6^ reads with an average read depth per sample of 143,102 ± 8,044. The 18S libraries resulted in the least number of paired reads, with 5.17x10^6^ reads and a read depth per sample of 101,928 ± 5,398.

Contamination levels in the sequencing negative controls and DNA extractions were low (**Supplemental Table 3**), representing between 0.002-0.013% of total reads, with the majority of the contamination sequences (>68%) coming from the extraction controls. While isolate sequences from the piglet fecal mock community appeared in the contamination sequences in very low abundance, the majority of the contaminants were not from isolates.

**Figure 2:**
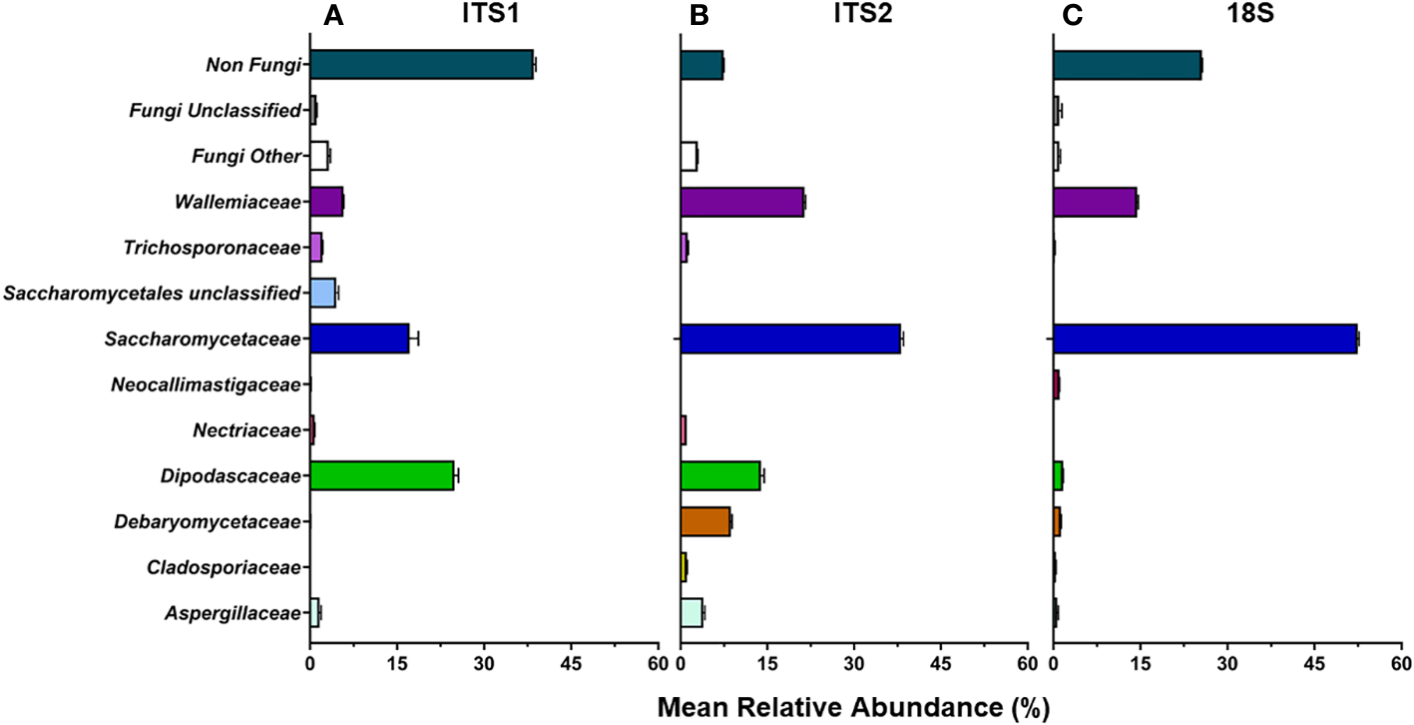
D24 fecal community. ITS1 **(A)**, ITS2 **(B)**, and 18S **(C)** primers were used to determine mean relative abundance of fungi found in post-weaning piglets (d24).

Of the ITS1, ITS2, and 18S libraries, ≥99.7% of reads for the Isolate, Mixed Mock, and ATCC Reference Standard communities were taxonomically identified as fungi using either UNITE ITS or SILVA 18S databases (**Table 4**) (**Supplemental Table 3**). For the Fecal community, 92.5% ± 0.8 of reads per sample were identified as fungus from the ITS2 library (162 total fungal ASVs), while only 74.3% ± 0.5 of the reads in the 18S library (57 total fungal ASVs) and 61.9% ± 0.7 reads in the ITS1 library (111 total fungal ASVs) were identified as fungus (**Figure 2**). This data supports the use of ITS2 for fungal taxa amplification in pig feces. Sequencing of this region resulted not only in the greatest sequencing yields and the highest number of identified ASVs, but also the lowest non-target amplification (**Supplemental Table 4**).

**Table 4:** Sequences identified as fungal from ITS1, ITS2, and 18S amplicon sequencing using the UNITE ITS or Silva 18S databases.

Sample	Description	ITS1 All	ITS1 Fungal	ITS1	ITS2 All	ITS2 Fungal	ITS2	18S All	18S Fungal	18S
		Sequences	Sequences	Sequences	% Fungus	Sequences	Sequences	% Fungus	Sequences	% Fungus
**ATCC-18Sa**	ATCC Reference Standard	216,347	216,347	100.0	151,428	151,428	100.0	29,926	29,831	99.7
**ATCC-18Sb**	ATCC Reference Standard	281,004	281,004	100.0	143,514	143,514	100.0	151*	110*	72.8*
**ATCC-18Sc**	ATCC Reference Standard	268,660	268,660	100.0	100,076	100,076	100.0	104,724	104,647	99.9
**ATCC2-18Sa**	ATCC Reference Standard	873,647	873,647	100.0	199,553	199,553	100.0	27,778	27,751	99.9
**ATCC2-18Sb**	ATCC Reference Standard	525,129	525,129	100.0	172,878	172,878	100.0	96,266	96,241	100.0
**ATCC2-18Sc**	ATCC Reference Standard	115,200	115,200	100.0	116,659	116,659	100.0	73,825	73,804	100.0
**C-18Sa**	C	321,844	321,844	100.0	110,226	110,226	100.0	66,710	66,710	100.0
**C-18Sb**	C	302,379	302,379	100.0	105,032	105,032	100.0	136,926	136,926	100.0
**C-18Sc**	C	334,917	334,917	100.0	138,004	138,004	100.0	154,817	154,817	100.0
**K-18Sa**	K	197,052	197,045	100.0	102,368	102,368	100.0	80,883	80,883	100.0
**K-18Sb**	K	175,115	175,108	100.0	151,050	151,050	100.0	109,118	109,118	100.0
**K-18Sc**	K	240,375	240,375	100.0	59,111	59,111	100.0	91,949	91,949	100.0
**M-18Sa**	M	103,287	103,121	99.8	46,674	46,674	100.0	84,989	84,989	100.0
**M-18Sb**	M	123,107	122,867	99.8	62,931	62,931	100.0	114,244	114,244	100.0
**M-18Sc**	M	75,192	75,064	99.8	74,567	74,567	100.0	108,347	108,347	100.0
**P-18Sa**	P	282,184	282,184	100.0	201,596	201,596	100.0	143,021	143,021	100.0
**P-18Sb**	P	449,849	449,849	100.0	85,021	85,021	100.0	41,668	41,668	100.0
**P-18Sc**	P	291,850	291,850	100.0	129,177	129,177	100.0	134,574	134,574	100.0
**T-18Sa**	T	201,244	201,244	100.0	157,752	157,752	100.0	81,949	81,949	100.0
**T-18Sb**	T	228,066	228,066	100.0	182,574	182,574	100.0	100,822	100,818	100.0
**T-18Sc**	T	258,368	258,368	100.0	138,499	138,499	100.0	59,965	59,962	100.0
**MixA-18Sa**	KCTPM	138,282	138,282	100.0	130,129	130,129	100.0	110,821	110,821	100.0
**MixA-18Sb**	KCTPM	292,101	292,101	100.0	169,426	169,426	100.0	125,302	125,297	100.0
**MixA-18Sc**	KCTPM	210,269	210,269	100.0	110,507	110,507	100.0	102,825	102,825	100.0
**MixB-18Sa**	KC	136,187	136,187	100.0	132,536	132,536	100.0	96,232	96,225	100.0
**MixB-18Sb**	KC	139,152	139,152	100.0	141,500	141,500	100.0	97,744	97,738	100.0
**MixB-18Sc**	KC	152,675	152,675	100.0	94,197	94,197	100.0	144,150	144,145	100.0
**MixC-18Sa**	KT	246,612	246,612	100.0	115,898	115,898	100.0	116,841	116,836	100.0
**MixC-18Sb**	KT	233,873	233,873	100.0	130,638	130,638	100.0	111,852	111,852	100.0
**MixC-18Sc**	KT	232,672	232,672	100.0	156,547	156,547	100.0	112,806	112,799	100.0
**MixD-18Sa**	D24	8,413	5,104	**60.7**	142,635	131,924	**92.5**	23,292	17,522	**75.2**
**MixD-18Sb**	D24	7,305	4,523	**61.9**	104,004	96,379	**92.7**	29,260	21,689	**74.1**
**MixD-18Sc**	D24	7,307	4,601	**63.0**	74,674	68,991	**92.4**	47,743	35,145	**73.6**
**MixF-18Sa**	KP	139,972	139,972	100.0	162,227	162,227	100.0	98,435	98,435	100.0
**MixF-18Sb**	KP	177,714	177,714	100.0	138,624	138,624	100.0	77,041	77,041	100.0
**MixF-18Sc**	KP	168,201	168,201	100.0	165,831	165,831	100.0	109,204	109,204	100.0
**MixG-18Sa**	KM	75,977	75,977	100.0	64,326	64,326	100.0	158,573	158,566	100.0
**MixG-18Sb**	KM	49,379	49,379	100.0	88,947	88,947	100.0	195,678	195,678	100.0
**MixG-18Sc**	KM	67,565	67,565	100.0	72,994	72,994	100.0	237,263	237,257	100.0
**MixH-18Sa**	KCT	206,400	206,400	100.0	209,313	209,313	100.0	87,890	87,881	100.0
**MixH-18Sb**	KCT	146,795	146,795	100.0	151,530	151,530	100.0	122,291	122,285	100.0
**MixH-18Sc**	KCT	166,199	166,199	100.0	238,977	238,977	100.0	143,523	143,515	100.0
**MixI-18Sa**	KCM	267,948	267,948	100.0	324,762	324,762	100.0	179,015	179,005	100.0
**MixI-18Sb**	KCM	220,305	220,305	100.0	352,179	352,179	100.0	119,578	119,563	100.0
**MixI-18Sc**	KCM	229,932	229,932	100.0	195,562	195,562	100.0	138,472	138,451	100.0
**MixJ-18Sa**	KTP	378,878	378,876	100.0	62,507	62,507	100.0	53,611	53,595	100.0
**MixJ-18Sb**	KTP	247,084	247,084	100.0	239,078	239,078	100.0	96,828	96,819	100.0
**MixJ-18Sc**	KTP	362,214	362,214	100.0	223,709	223,709	100.0	84,090	84,075	100.0
**MixK-18Sa**	KTM	282,468	282,468	100.0	191,181	191,181	100.0	90,063	90,063	100.0
**MixK-18Sb**	KTM	375,484	375,484	100.0	199,343	199,343	100.0	71,909	71,909	100.0
**MixK-18Sc**	KTM	292,127	292,127	100.0	77,309	77,309	100.0	119,594	119,594	100.0
**MixL-18Sa**	KPM	297,558	297,558	100.0	193,837	193,837	100.0	125,864	125,828	100.0
**MixL-18Sb**	KPM	174,932	174,932	100.0	79,975	79,975	100.0	95,827	95,813	100.0
**MixL-18Sc**	KPM	222,200	222,200	100.0	140,314	140,314	100.0	112,623	112,613	100.0
**MixM-18Sa**	KCP	238,044	238,044	100.0	145,369	145,369	100.0	75,786	75,780	100.0
**MixM-18Sb**	KCP	161,580	161,580	100.0	134,908	134,908	100.0	78,079	78,073	100.0
**MixM-18Sc**	KCP	403,014	403,014	100.0	172,619	172,619	100.0	75,374	75,374	100.0
*Excluded from analysis										

This table represents the results for the triplicate sequencing runs for each of the mock communities.

The names of the samples are in bold.

Next, we assessed total community ASV lengths (**Supplemental Table 4**). The fungal ASV and amplicon lengths varied significantly between the ITS1, ITS2, and 18S libraries. 111 fungal ASVs with a mean length of 239 bp ± 7.9 bp (min = 106, max = 478) were identified in the ITS1 library. In the ITS2 library, 162 fungal ASVs with a mean length of 307 bp ± 5.0 (min = 101, max = 436) were identified and 57 fungal ASVs with a mean length of 324 bp ± 7.6 (min = 196, max = 535) in the 18S library were identified.

The ITS1 ASV libraries from individual isolates had the greatest range in ASV lengths from 154 bp (*P. fermentens*) to 395 bp (*K. slooffiae*), while the 18S library had the smallest range from 312 bp (*P. fermentens*) to 335 bp (*L. corymbifera*) (**Supplemental Table 5**). With the exception of *K. slooffiae*, respective ITS1 ASV lengths were between 54 and 119 bp shorter than ITS2 ASV lengths. ASVs for *K. slooffiae* ITS2 was 5-6 bp longer than ITS1.

### Taxonomic classification of isolates, mixed, and ATCC Reference Standard ASVs

Taxonomic classifications of Isolate, Mixed, and ATCC Reference Standard ASVs were determined using a trained Naïve Bayes classifier with the UNITE v 8 database for ITS1 and ITS2 libraries and the SILVA 138 database for the 18S library (**Table 5**). All ASVs were additionally identified using BLAST (blastn) against the NCBI nt database (selecting for TYPE material, specimens used to originally describe species).

**Table 5:** Single species sequencing results.

	*Kazachstania slooffiae*	*Candida albicans*	*Trichosporon*	*Pichia*	*Mucor*
***Kazachstania***	99.71	0.3	0.3	0	0.27
***Candida albicans***	0	99.45	0.18	0.08	0.17
***Trichosporon***	0.23	0.25	99.52	0.06	0
***Pichia***	0	0	0	0	0
***Mucor***	0	0	0	0	0
*Fusarium*	0.05	0	0	0.01	0
*Candida intermedia*	0.01	0.01	0	0	0
*Dipodascus australiensis*	0	0	0	0.01	0.03
*Diaporthe longicolla*	0	0	0	0.01	0
*Lichtheimia corymbifera*	0	0	0	0.23	99.45
*Saccharomycetales*	0	0	0	99.6	0.09
**% Relative Abundance**	**100**	**100**	**100**	**100**	**100**

This table demonstrates the relative abundance of the ASVs identified in each of the 5 isolate samples sequenced on the Illumina MiSeq platform; Kazachstania slooffiae, Candida albicans, Trichosporon, Pichia, and Mucor. Total relative abundance is in bold.

**Figure 3:**
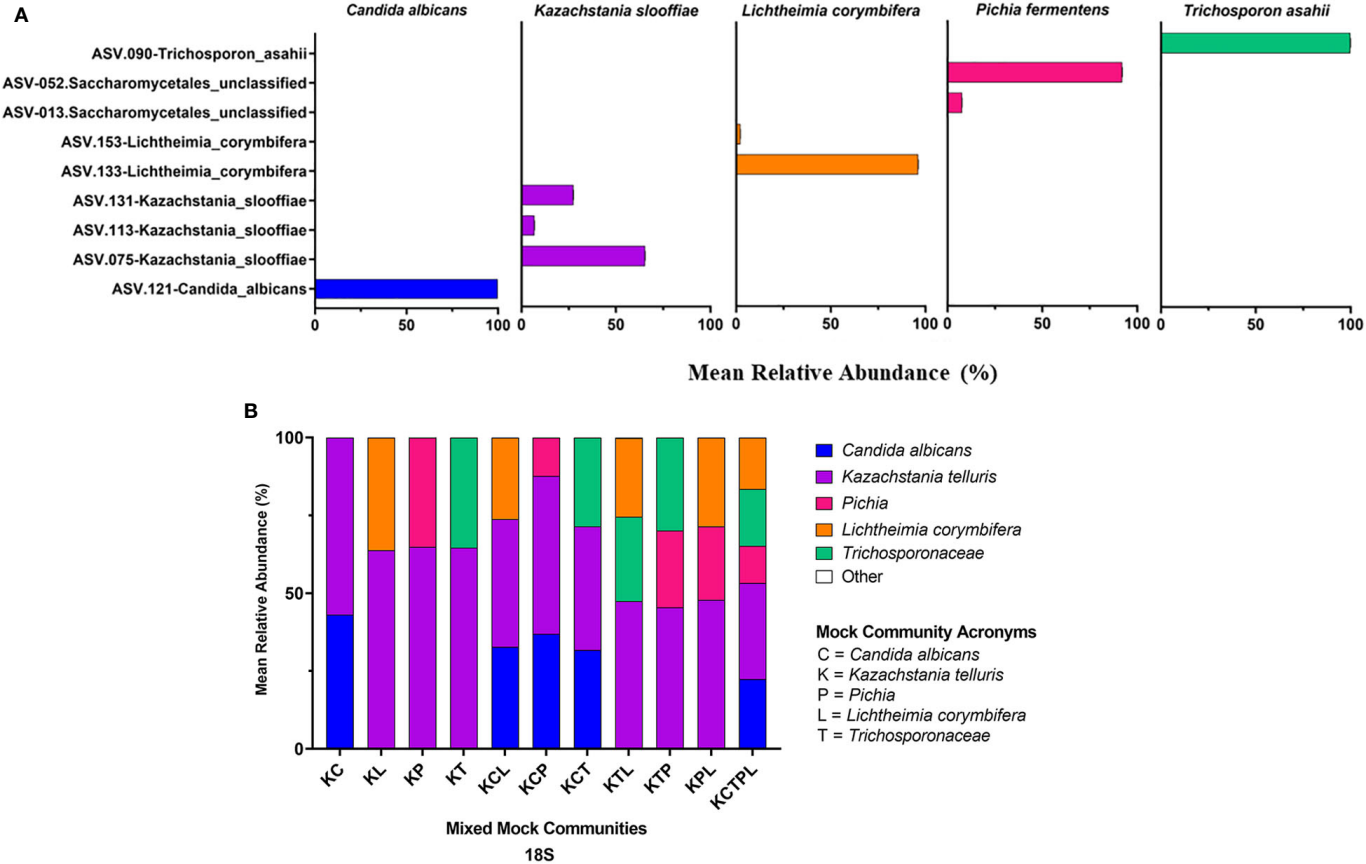
ITS1 Sequencing Results. ITS primers were used to sequence individual fungal isolates; *C albicans, K slooffiae, L. corymbifera, P. fermentans*, and *T. asahii* **(A)** or mock communities ranging from 2 isolate combinations up to all 5 in one mock community **(B)**. Each mock community is represented on the x axis with an acronym representing the fungi in the mock community. C, *Candida albicans*; K, *Kazachstania telluris*; P, *Pichia*; L, *Lichtheimia corymbifera*; and T, *Trichosporonaceae*. Combinations are represented with the first letter of each fungus found in each mock community, for example, KC, *Kazachstania* and *Candida*. Negative controls for PCR reactions are found in the supplemental data.

**Figure 4:**
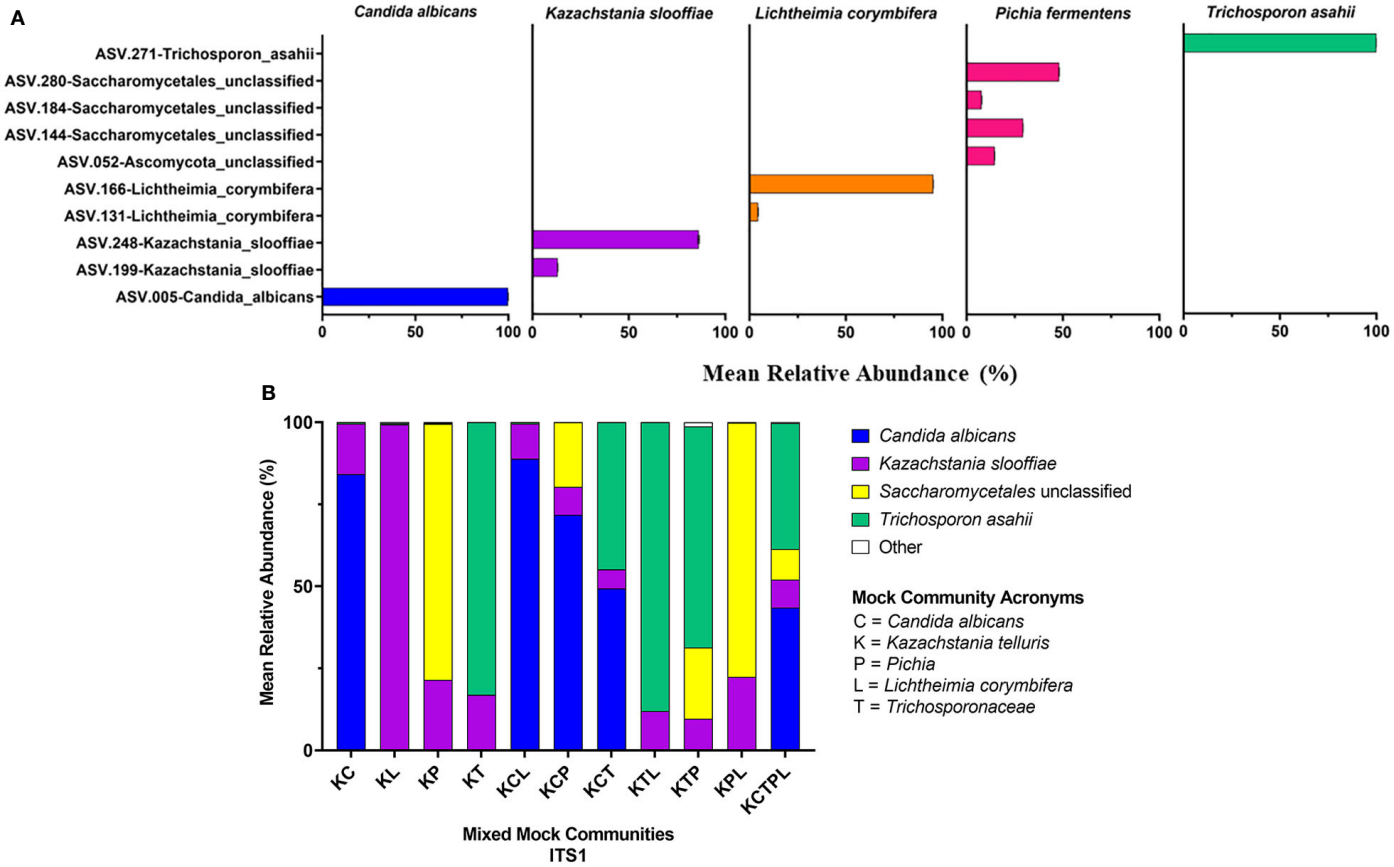
ITS2 Sequencing Results. ITS primers were used to sequence individual fungal isolates, *C albicans, K slooffiae, L. corymbifera, P. fermentans*, and *T. asahii* **(A)** or mock communities ranging from 2 isolate combinations up to all 5 in one mock community **(B)**. Each mock community is represented on the x axis with an acronym representing the fungi in the mock community. C, *Candida albicans*; K, *Kazachstania telluris*; P, *Pichia*; L, *Lichtheimia corymbifera*, and T, *Trichosporonaceae*. Combinations are represented with the first letter of each fungus found in each mock community, for example, KC, *Kazachstania* and *Candida*.

**Figure 5:**
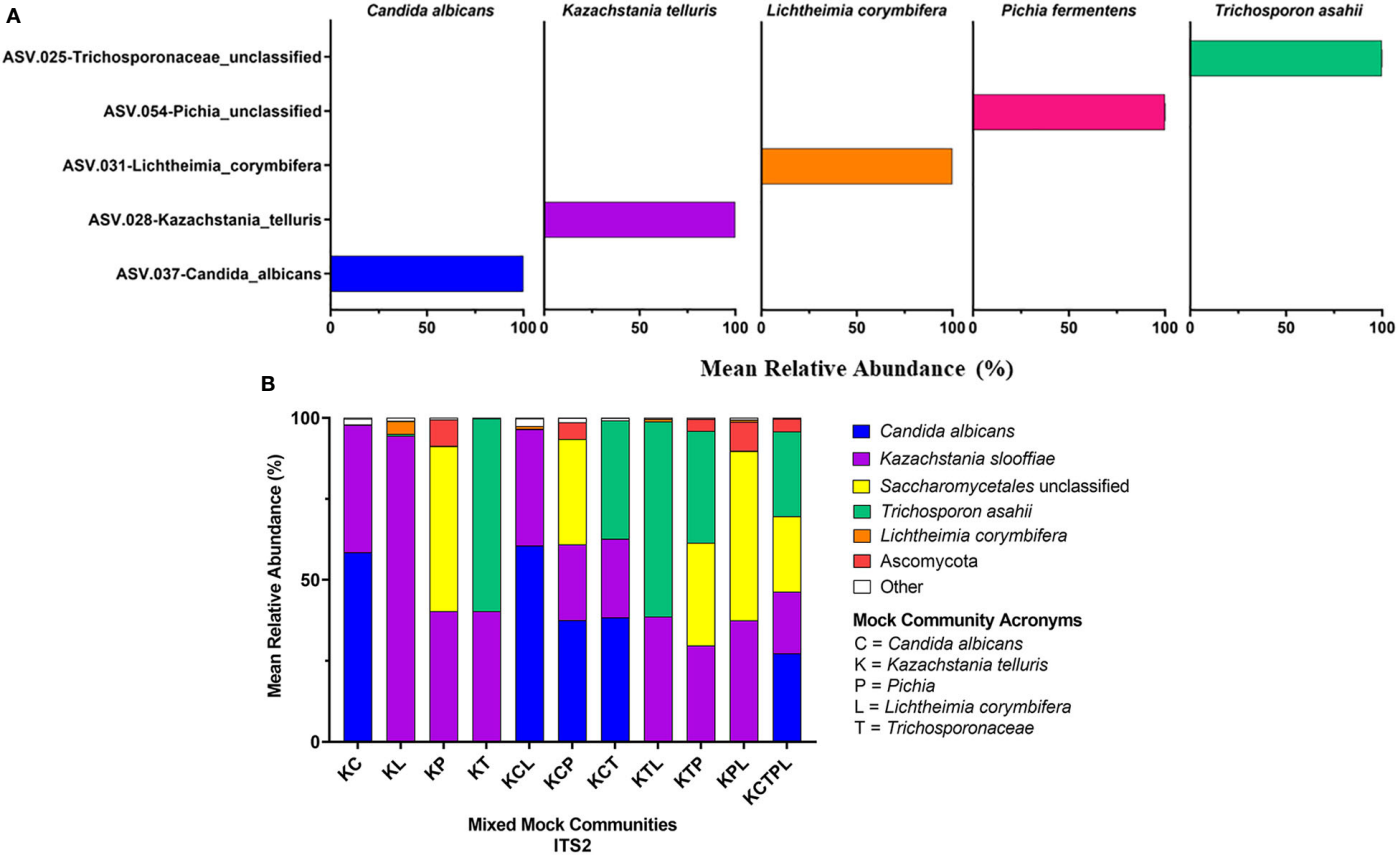
18S Sequencing Results. 18S primers were used to sequence individual fungal isolates, *C albicans, K slooffiae, L. corymbifera, P. fermentans*, and *T. asahii* **(A)** or mock communities ranging from 2 isolate combinations up to all 5 in one mock community **(B)**. Each mock community is represented on the x axis with an acronym representing the fungi in the mock community. C = *Candida albicans*, K = *Kazachstania telluris*, P = *Pichia*, L = *Lichtheimia corymbifera*, and T = *Trichosporonaceae*. Combinations are represented with the first letter of each fungus found in each mock community, for example, KC = *Kazachstania* and *Candida*.

Of the amplicons generated from the five individually sequenced isolates, the 18S amplicons were the only ones that were classified into a single ASV in all five isolates. The ITS1 and ITS2 amplicons from *C. albicans* and *T. asahaii* were also classified into a single ASV, while those from *K. slooffiae*, *L. corymbifera*, and *P. fermentens* were classified into 2-4 ASVs each (**Figures 3A**, **4A**, **5A**).

*Kazachstania slooffiae.* The ASVs from the *K. slooffiae* isolates were correctly identified in the ITS1 and ITS2 libraries by the UNITE database and using BLAST. In the 18S library, however; the *K. slooffiae* isolate was taxonomically identified as the closely related *K. telluris* species due to the absence of *K. slooffiae* in the SILVA database and NCBI nr/nt database for the 18S gene.

*Candida albicans* & *Lichtheimia. corymbifera.* The ASVs associated with the *C. albicans* and *L. corymbifera* isolates were correctly identified in all three libraries by both BLAST and the UNITE/SILVA databases. However, in the 18S library, BLAST returned other similarly close fungal matches in addition to *L. corymbifera* for the *L. corymbifera* isolate, including *L. (Absidia) blakesleeana*.

*Trichosporon asahaii.* The ASVs associated with the *T. asahaii* isolate were correctly identified in both the ITS1 and ITS2 libraries by the UNITE database, but only classified down to the family level (*Trichosporonaceae*) in the 18S library by the SILVA database. Additionally, the closest BLAST match to the *T. asahii* ASV from the ITS1 library was *T. faecale*, while several different fungal species were similarly closely matched to the ASV from the 18S library, including genera *Pascua*, *Cryptococcus*, and *Apiotrichum*.

*Pichia fermentens.* None of the ASVs associated with *P. fermentens* isolate were identified at the species level in any of the libraries by the UNITE/SILVA databases. In the 18S library, *P. fermentens* was correctly identified down to the genus level, while only identified to the order level Saccharomycetales in the ITS1 and Saccharomycetales or Ascomycota ITS2 libraries. However, BLAST results of the ASVs from all three libraries accurately identified the isolate as *P. fermentens*.

ATCC Reference Standard community. Using the UNITE database, 6 of the isolates found in the ATCC Reference Standard community were correctly identified in the ITS2 library down to the species level, while 4 were correctly classified at the genus level, with 3 having improper species identification (**Figures 3B**, **4B**, **5B** and **Table 5**). In the ITS1 library, 3 were correctly identified at the species level, 6 at the genus level (3 having improper species identifications), and one isolate could not be taxonomically identified (*Candida glabrata*) from the library. The 18S library with the SILVA database had the poorest taxonomic classification, with only 2 members having the proper genus-species identification, 5 classified down to the genus level, and 3 at the family or order level.

*Mixed communities.* The ITS1 and ITS2 workflows identified all species except for *L. corymbifera* in all samples, but the ITS2 workflow did identify this species in one of five mixed communities. Similar to the single isolate samples, *P. fermentens* was only identified to the order level Saccharomycetales in the ITS1 and Saccharomycetales or Ascomycota ITS2 libraries. The 18S + SILVA workflow *T. asahaii* was identified only to the family level (*Trichosporonaceae*), and *K. slooffiae* was misidentified as *K. telluris*.

### Abundance estimates of ATCC Reference Standard and mixed mock communities

The relative abundances of ASVs identified from isolates were determined for each of the constructed and ATCC Reference Standard mock communities (**Figures 3**–**5** and **Supplemental Tables 5–8**).

*ITS1*. *K. slooffiae* was under-represented in relative abundance in the mixed mock communities, while *C. albicans and T. asahii* were over-represented (**Figure 3**). The ASV amplified from the *P. fermentans* isolate (*Saccharomycetales* unclassified) was over-represented in mock communities KP and KPM, but under-represented in mock communities containing *C. albicans* or *T. asahii*. *L. corymbifera* was almost entirely absent in the ITS1 library, with <1% relative abundance of reads in the mixed mock communities containing the isolate.

*ITS2*. *C. albicans and T. asahii* were slightly over-represented in relative abundance in the ITS2 library for dual mixed communities, but less than ITS1 (**Figure 4**). For mixed communities KCP, KCT, KTP containing 3 isolates, *C. albicans* and *T. asahii* were close to the expected abundances. *K. slooffiae* was only slightly under-represented in the ITS2 library. *L. corymbifera* was greatly under-represented in the ITS2 library for all mixed communities in which it was present, with only the KM library having a mean relative abundance of *L. corymbifera >*1% (3.88% ± 0.25).

*18S.* The 18S library was the only library in which *L. corymbifera* ASVs were successfully represented, although still under-represented compared to the other isolates (**Figure 5**). All isolates were slightly under-represented in the mix mock communities except for *K. slooffiae*, which was slightly over-represented. In general, the 18S library was the closest representation of the original mock communities when considering all isolates used in this study.

**Figure 6:**
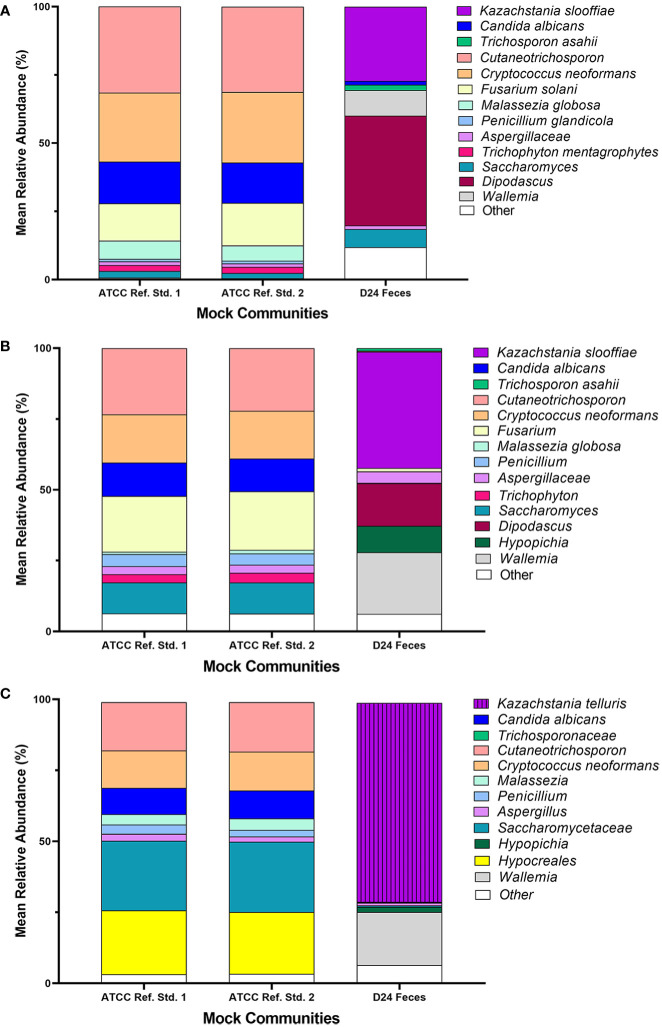
**(A)** ATCC Reference Standard Sequencing Results ITS1. Technical replicates of the ATCC mock fungal community (ATCC Reference Standard 1 and ATCC Reference Standard 2) and DNA extracted from piglet feces (D24 feces) were sequenced on a MiSeq Illumina platform utilizing ITS1 primers. The UNITE database was utilized to identify taxonomy of the population. **(B)** ATCC Reference Standard Sequencing Results ITS2. Technical replicates of the ATCC mock fungal community (ATCC Reference Standard 1 and ATCC Reference Standard 2) and DNA extracted from piglet feces (D24 feces) were sequenced on a MiSeq Illumina platform utilizing ITS2 primers. The UNITE database was utilized to identify taxonomy of the population. **(C)**. ATCC Reference Standard Sequencing Results 18S. Technical replicates of the ATCC mock fungal community (ATCC Reference Standard 1 and ATCC Reference Standard 2) and DNA extracted from piglet feces (D24 feces) were sequenced on a MiSeq Illumina platform utilizing 18S primers. The SILVA database was utilized to identify taxonomy of the population.

ATCC Reference Standard mock community*. A. fumigatus*, *T. interdigitale*, and *P. chrysogenum* were under-represented in relative abundances in the ATCC Reference Standard communities with relative abundances <50% the expected composition (**Figure 6** and **Table 5**). In contrast, *C. dermatis* was more than ~1.7-3.2x over-represented in all libraries. Only *C. albicans* was close to the expected composition by relative abundance in the libraries, ranging from ~15.0% in the 18S library to ~9.5% in the ITS2 library. Of the 3 libraries, the ITS2 library was the closest match in the distribution of relative abundances to the original ATCC Reference Standard mixed mock community (cumulative difference of 64.89), followed closely by 18S (cumulative difference of 69.6), with ITS1 the least similar (cumulative difference of 93.8). The poorest represented isolates from the ATCC Reference Standard mock community were *P. glandicola* and *C. glabrata* in the ITS1 library and *M. globosa* in the ITS2 library with mean relative abundances of ~1.0%

### Fecal community composition

For the Fecal community, the ITS1 library had the highest number of non-target ASVs (38.5%) followed by the 18S library (25.6%) (**Figure 2A**). The ITS2 library had the least number of non-target ASVs (7.5%). Of the non-target ASVs in the Fecal community, 17.2% ± 0.4 matched to *Blastocystis* sp. in the ITS1 library, 12.1% ± 0.1 matched to Glycine max in the ITS2 library, and 86.25% ± 0.1 matched to *Blastocystis* sp. in the 18S library using BLAST with the NCBI nt database.

At the family level, ITS2 overall showed more taxonomic richness (numerical count of different species) in the Fecal community compared to the other 2 libraries (**Figure 2B**). At the ASV level, ITS2 had the highest number of ASVs with 51 fungal ASVs found. 47 fungal ASVs were identified in the ITS1 library and 31 fungal ASVs were identified in the 18S library.

The ASVs from the *L. corymbifera* isolate were not detected in any of the Fecal samples for ITS1, ITS2 or 18S. ASVs from isolates *T. asahii*, *C. albicans*, *P. fermentens*, and *K. slooffiae* were identified in the ITS1 library for D24, while only ASVs from isolates from *K. slooffiae* and *T. asahii* were detected in the ITS2 and 18S library, with *T. asahii* representing <1% abundance in the 18S library.

### Copy number bias

**Figure 7:**
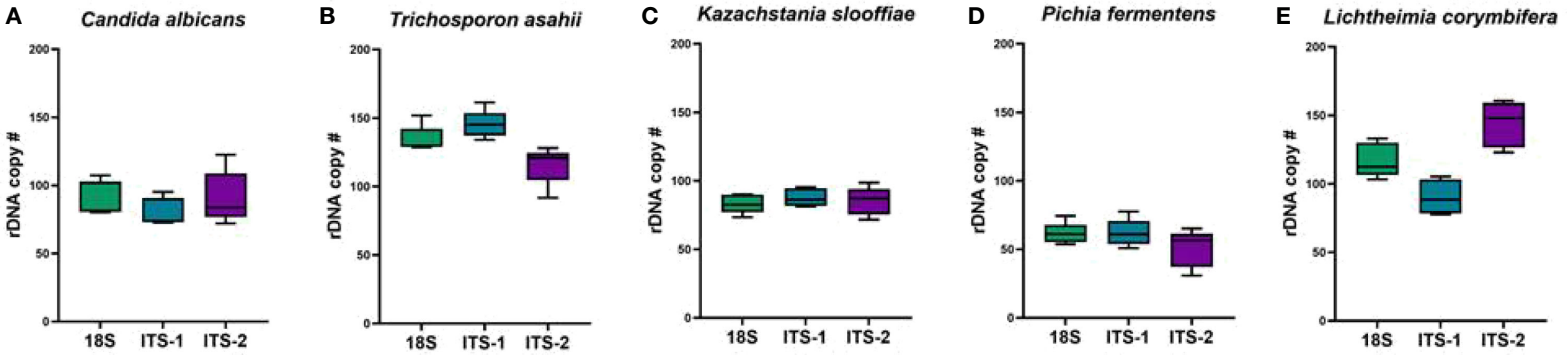
Estimated rDNA copy numbers. Primers targeting 18S, ITS1, and ITS2 were analyzed for copy number in each of the five fungal species utilized in the mock community. **(A)** *Candida albicans*, **(B)** *Trichosporon asahii*, **(C)** *Kazachstania slooffiae*, **(D)** *Pichia fermentans* and **(E)** *Lichtheimia corymbifera*.

We estimated the copy number of each of the ITS or 18S regions within each fungal cell. To estimate the copy number of the ribosomal RNA gene within the genome of each fungal strain, qPCR assays were designed based on the genome sequence data available for each strain or the most closely related strain available. The copy number for each strain in the mock community demonstrated variability based on amplification region (**Figure 7**). While *K. slooffiae* demonstrated the most stable number of copy numbers of the five species analyzed (**Figure 7C**) the most variability was seen in *L. corymbifera* (**Figure 7E**). *T. asahii* had the most copy numbers on average across region amplified (**Figure 7B**) and *P. fermentans* displayed the fewest copy numbers (**Figure 7D**).

## Discussion

Fungi are considered part of the rare biosphere in the gut, due to their numerical inferiority compared to bacteria, but still play an important role in host health (Huffnagle and Noverr, 2013). Yet, relatively little information is available regarding the mycobiome, particularly in agricultural animals. Previous work suggests that the porcine mycobiome has a pattern of α-diversity that is distinct than that of the human gut bacteriome (Arfken et al., 2020), highlighting the need for more animal mycobiome studies. Fungal sequencing has lagged in part due to difficulties in DNA extraction protocols, primer design, database inaccuracies and missing data (Huffnagle and Noverr, 2013; Huseyin et al., 2017; Thielemann et al., 2022). Thus, technical studies which determine the most accurate sequencing protocols are needed to reduce biases and incorrect research results. In this study, we created a porcine fungal mock community for use in understanding potential biases in an Illumina MiSeq sequencing workflow. We analyzed this community, along with a commercial mock community, piglet fecal samples, and individual fungal isolates from piglet feces using a workflow which varied in the gene region targeted and the reference database.

We did not determine that a single marker and database combination correctly identified all species in the mock communities. In Mixed communities, ITS marker workflows correctly identified *K. slooffiae*, *C. albicans*, and *T. asahaii*, but *L. corymbifera* was not identified except in a single community by the ITS2 workflow, and *P. fermentens* was only identified to the order or phylum level. In contrast, the 18S + SILVA workflow correctly identified *C. albicans, L. corymbifera*, and *P. fermentens*, but *K. slooffiae* was identified as *K. telluris*, and *T. asahaii* was only identified to the family level. In ATCC Reference Standard communities consisting of 10 environmental species, workflow performance could be ranked as ITS2 > ITS1 > 18S, with the number of correct species identified as 6,4, and 2, respectively. In the Fecal community, the greatest percentage of fungal taxa were identified from ITS2 amplicons (92.5% ± 0.8), while only 74.3% ± 0.5 of the reads in the 18S library were fungi, and 61.9% ± 0.7 in the ITS1 library. The absence of *L. corymbifera* in Mixed communities is likely due to unequal amplification of species by ITS primers, as the species was correctly identified in the single isolate communities. The lack of detection of *L. corymbifera* is especially problematic in pig studies as it is often found in pre-weanling piglets. *M. globera* (*Mucor* is synonymous with *Corymbifera*) was not amplified by ITS2 primers in the ATCC Reference Standard community, although it was amplified in the 18S dataset, suggesting a potential ITS2 primer design issue for this genus.

The preferential amplification of certain species by some markers and not others has been previously described (Karlsson et al., 2017; Walder et al., 2017; Bakker, 2018) and Nilsson et al. demonstrated that primers are the driving factor in which fungal species will be identified in samples (Nilsson et al., 2019). In studies of environmental fungi, some primers have mismatches to certain classes of fungi including Saccharomycetes and Chytridiomycota. The ITS1 forward primer utilized in multiple studies found a 3’ terminal mismatch that lowers the efficacy of amplifying these classes, which provides ITS2 primers an advantage for sequencing Saccharomycetes (Tedersoo et al., 2015; Mbareche et al., 2020). One factor accounting for taxonomic identification differences between ITS1 and ITS2 primers is the presence of introns within primer sites. One documented phylum effected by intron presence is Ascomycota (Bhattacharya et al., 2000; Perotto et al., 2000). To avoid biases due to differential amplification, and increase taxonomic breadth of identified taxa, a dual-marker approach using both 18S and ITS has been proposed (Raja et al., 2017; D’Andreano et al., 2021). Given the complimentary species identifications observed here among the markers, this approach may be fruitful for porcine mycobiome studies.

Fungal marker gene lengths, particularly those of ITS regions, are variable, and marker length may affect both taxonomic assignments and abundance estimates. We detected a range of lengths in all three amplicons (**Supplemental Table 5**), which is in line with previous studies. For example, in the Ascomycota and Basidiomycota, complete ITS sequences (ITS1, ITS2, + 5.8) range in length from 600 to 900 bp (Toju et al., 2012). Longer amplicons may include a greater percentage of low-quality sequences compared to shorter amplicons, due either to quality reduction at the end of the read due to polymerase fall-off, or through a reduction in paired-end ligation which can also decrease sequence quality (Kircher et al., 2011; Rae et al., 2015; Amarasinghe et al., 2020). Furthermore, longer reads may be amplified at lower rates than shorter amplicons as some polymerases preferentially amplify shorter fragments (Reich and Labes, 2017). Some ITS2 primers, including the ones utilized in this study, include a portion of the 5.8 rRNA gene in the amplification of the ITS2 region, which results in a longer amplicon (Lindahl et al., 2013; Tedersoo et al., 2015; Tedersoo and Lindahl, 2016). Here, it is unclear whether longer sequences led to biases during amplification or sequencing. Suggested methods to avoid such complications are to dilute fungal DNA, keep PCR amplification cycle numbers low, and utilize high-fidelity polymerases with low GC bias (D'Amore et al., 2016; Gohl et al., 2016; Castle et al., 2018; Nilsson et al., 2019), thus future studies could include these steps.

Different marker genes may yield different numbers of ASVs, as was the case in our study. ITS2 sequences yielded the highest fungal richness as defined by the highest number of ASVs, 162, while 18S amplicons were the most conservative in fungal richness with 57 ASVs. The ITS region is more variable than 18S, and therefore it is not surprising that ITS markers yielded a greater number of ASVs (Nilsson et al., 2019). However, a greater number of ASVs may not indicate increased taxonomic resolution if observed variability does not correlate with new species or subspecies boundaries. Furthermore, sequence conservation can vary even within marker regions, and the selection of different primers amplifying these regions may impact amplicon variability, and thus the number of identified ASVs. Recently, Mbareche et al. assessed the performance of ITS1 and ITS2 primers in amplifying fungal species from bioaerosols and demonstrated that their chosen ITS1-targeting primers were most effective at detecting the most richness and taxonomic coverage (Mbareche et al., 2020).

The extent of off-target amplification varies across markers, with 92.5% ± 0.8 of ITS2 reads mapping to fungi, 74.3% ± 0.5 of 18S reads and 61.9% ± 0.7 of ITS1 reads. These results are in line with previous studies demonstrating the amplification of non-fungal taxa. Primers for ITS amplify protozoa such as *Blastocystis* (Martin and Rygiewicz, 2005; Bellemain et al., 2010; AbuOdeh et al., 2016; Stensvold and Clark, 2016) and Ciliophora (Summers and Arfken 2022), some bacterial species such as *Escherichia coli* and Bacteroides (Summers and Arfken 2022) and some Plantae, presumably from food sources (Summers and Arfken 2022). Off-target amplified species differ depending on whether ITS1 and ITS2 are employed (Summers and Arfken 2022). Kounosu et al., reported that commonly used 18S primers amplified bacterial 16S genes (Kounosu et al., 2019), while another found that their tested 18S markers did not amplify prokaryotic species (Hadziavdic et al., 2014). Non-fungi eukaryotic organisms may also be amplified by 18S primers (Liu et al., 2019).

Abundance estimates may also be affected by the choice of marker. In our study, relative abundance values derived from 18S amplicons best represented abundances of all fungal Mixed mock communities due in part to the accurate representation of *L. corymbifera* and *Pichia* (**Figures 3**–**5**). Abundances derived from ITS data were less accurate than those of 18S, with ITS1 data showing the lowest accuracy (**Figures 3**–**5**). Although input DNA for all species was equivalent, ITS based abundance profiles rarely represented each taxon in equal proportions. However, ITS primers most accurately represented the abundances of ATCC Reference Standard mock community species, with the exception of *M. globosa*. Regardless of primer choice, *Malassezia* and *Aspergillus*, both members of the Ascomycota, were underrepresented in all ATCC Reference Standard community samples (**Figures 6A–C**). It is not possible to determine which markers yielded the most accurate abundances in fecal samples, since real abundances are unknown. However, our previous culture-based studies have detected moderate levels of *Aspergillus* in piglet fecal samples, and here, only ITS2 detected a moderate level of *Aspergillus* (**Figure 2**).

In addition to marker gene selection, database choice is a critical factor in mycobiome analyses. The development of databases such as SILVA for 18S data and UNITE for ITS data has greatly facilitated the study of fungi, but additional effort is needed to develop these resources. Not all taxonomic groups are represented in the databases, which can result in an amplicon being either unclassified or misclassified. For example, *K. slooffiae* was identified as *K. telluris* in our 18S dataset, because *K. slooffiae* is not present in SILVA. Sequencing or other errors within database sequences can further introduce errors during the classification process. Lastly, sequences may be assigned incorrect taxonomic labels. Incorrect classification of species may also result in erroneous downstream interpretation of data. For example, estimates of community richness may be skewed. Current studies indicate that healthy animals have lower fungal α-diversity in the gut than bacterial α-diversity (reviewed in (Summers and Arfken, 2022)), but these observations may be due to missing database sequences. Thus, increasing the number and quality of database sequences should be a priority, and sequencing of both marker genes and whole genomes of uncharacterized fungi will advance these efforts.

In our study, we used two primary methods for fungal taxonomic identification: (1) a QIIME2 trained Naïve Bayes classifier with either the UNITE ITS or SILVA 18S database and a (2) traditional blastn search with the NCBI nr/nt database. The UNITE ITS classifications using a trained Naïve Bayes classifier in QIIME2 were more accurate than those based on SILVA 18S classification from *Trichosporon* and *Kazachstania*, but the trend was opposite for *Pichia*. The UNITE database was developed and is maintained specifically for the classification of fungi and eukaryotic organisms based on the ITS region, and thus, is likely more reliable for the most current and high-level fungal identification. While we selected to choose taxonomic workflows that were most often used in mycobiome analyses, running blastn against a stand-alone UNITE database with *P. fermentans* yielded the correct taxonomical identification, suggesting that the issue lies in the QIIME 2 classifier or taxonomic assignment, rather than the database. Search on BLAST with the NCBI nr/nt database were generally accurate for most of the fungal genera amplified with ITS1 and ITS2. The only exception was ITS1 *T. asahii*. The NCBI nr/nt database was not as useful for identifying 18S amplicons, however, due to multiple close database matches for isolates such as those closely related to Trichosporon and lacking representative sequences in the database, such as those for *K. slooffiae*. Based on our findings, the UNITE database targeting the ITS gene region is likely the best database for high-level fungal taxonomic identification. However, some fungal species may require more than one classifier/search method or require an additional database for accurate fungal classification.

Copy numbers were estimated for each fungal isolate by qPCR assays as previously published (Bakker, 2018). Each of the five isolates demonstrated variability in copy number for each amplification region. Overall, *K. slooffiae* had the most consistent copy numbers across 18S, ITS1, and ITS2 while *L. corymbifera* demonstrated the most variability. The fungal isolate with the most copy numbers across region amplified on average was *T. asahii* and *P. fermentans* had the fewest copy numbers. While *T. asahii* had the most copy numbers of the ITS1 region and outperformed *K. slooffiae* and *L. corymbifera* in the Mixed mock communities, it did not outperform *C. albicans*, suggesting that copy number does not result in a significant bias in our study. The level of *T. asahii* was not overabundant for ITS2 or 18S. While our copy number calculations are an estimate, we do not demonstrate correlation with copy number and abundances for these five fungal isolates.

Previous studies have demonstrated that *K. slooffiae* is the predominant fungi found in the GI tract and feces of post-weaning piglets (Urubschurov et al., 2008; Urubschurov et al., 2011; Urubschurov et al., 2017; Urubschurov et al., 2018; Arfken et al., 2019; Summers et al., 2019), and its genome contains multiple copies of ITS1 and ITS2 (Davies et al., 2021). This study indirectly assessed if the prevalence of *K. slooffiae* is an artifact of fungal sequencing biases found in mycobiome workflows. Our data demonstrate that 18S-based amplification with SILVA database analyses did not inflate or over-calculate the abundance of *K. slooffiae* in any of the mixed communities. Interestingly with ITS1 or ITS2 primers, *K. slooffiae* did not become overrepresented in any of the Mixed mock communities except for the dual community of mixed *K. slooffiae* and *L. corymbifera*, where *L. corymbifera* was not recognized in the workflow. Further, the estimated range of copy numbers of ITS1, ITS2, and 18S were lower than all other isolates except for *P. fermentans*. This data is especially useful in the porcine mycobiome field in confirming that the dominance of *K. slooffiae* in the swine gut is not over-assessed and may be, in fact, underestimated.

Our study has several caveats. Our study utilized five fungal isolates from piglet feces three days post-weaning. These isolates were chosen for their ability to be grown *in vitro* in the laboratory and therefore do not represent the entire fecal mycobiome of piglets (as represented in the fecal sample sequenced). We acknowledge that further isolates could be cultured, but our study was purposefully limited to five for simplicity of creating the Mixed mock communities. We cannot rule out the possibility that individual fungal colonies derived from piglet feces originated from a single, unique colony upon sequencing. In this case, the number of identified amplicons would be artificially inflated. It is also possible that sequencing errors resulted in a slight increase in the number of identified ASVs, as Illumina sequencing produces errors in 0.1% of nucleotides on average (Pfeiffer et al., 2018). Additionally, fungal genome size was not considered when creating mock communities, as genome sizes are unavailable or incomplete for all five of the fungal isolates and should be investigated further in the future. Genome size can be correlated with chromosome copy numbers in some species (Steenwyk and Rokas, 2018). However, to assess a potential copy number bias in this study, copy number ranges for 18S, ITS1, and ITS2 were estimated using qPCR assays (**Figure 7**). Future studies could utilize digital droplet PCR to perform a more targeted quantification or whole genome sequencing and annotation could result in more accurate copy number values.

Future studies should consider the utilization of multiple amplicon targets to determine whether this multi-step process improves the accuracy of taxonomic identifications and abundance estimates. A dual primer approach, using 18S and ITS primers have improved results of other fungal studies (Arfi et al., 2012; Reich and Labes, 2017; Banos et al., 2018). Additional studies should also address biases due to collection methods, storage conditions (10.1186/s40168-016-0186-x), DNA extraction technique (Enaud et al., 2018), as fungal cells walls are diverse and there is no current consensus on how to best extract DNA from all cells. Different primer pairs that target the same region could also be tested, as results may differ based on these choices.

This study demonstrates the importance of preliminary studies to evaluate biases present in amplicon-based sequencing workflows due to marker gene selection and database content. The use of appropriate mock community controls is a particularly useful strategy to differentiate true from biased results and should be utilized when possible. Ultimately, we did not identify one marker-database combination which outperformed all others, but instead observed strengths and weaknesses apparent in each combination tested. Thus, a dual-marker approach may be fruitful for pig mycobiome studies by sequencing 18S and ITS regions for optimal database information for taxonomic identifications. Careful consideration of the sample type and its potential fungal targeted populations are critical preliminary experimental design steps that assist in optimizing primer choices. Future studies of fungi in the gut, which minimize biases, will provide robust community profiles and provide opportunities to improve pig health through mycobiome manipulations.

## Data availability statement

The datasets presented in this study can be found in online repositories. The names of the repository/repositories and accession number(s) can be found in the article/**Supplementary Material**. All sequences are publicly available (BioProject PRJNA693350).

## Ethics statement

The animal study was reviewed and approved by USDA-ARS Institutional Animal Care and Use Committee of the Beltsville Agricultural Research Center. Written informed consent was obtained from the owners for the participation of their animals in this study.

## Author contributions

AA, JF, and KS are responsible for substantial contributions to the study conception and design. KS, NC, ND, OO, and DR were responsible for animal sampling, data acquisition, and preliminary data analyses. AA and KS were responsible for bioinformatics and analyses. AA, JF, CD, and KS were responsible for drafting of the manuscript and critical revision of the manuscript. All authors contributed to the article and approved the submitted version.
